# Self-Regulation, Cooperative Learning, and Academic Self-Efficacy: Interactions to Prevent School Failure

**DOI:** 10.3389/fpsyg.2017.00022

**Published:** 2017-01-19

**Authors:** Javier Fernandez-Rio, Jose A. Cecchini, Antonio Méndez-Gimenez, David Mendez-Alonso, Jose A. Prieto

**Affiliations:** ^1^Department of Educational Science, University of OviedoOviedo, Spain; ^2^Facultad Padre Ossó, University of OviedoOviedo, Spain

**Keywords:** secondary education, students at risk, clusters, academic self-efficacy, learning

## Abstract

Learning to learn and learning to cooperate are two important goals for individuals. Moreover, self regulation has been identified as fundamental to prevent school failure. The goal of the present study was to assess the interactions between self-regulated learning, cooperative learning and academic self-efficacy in secondary education students experiencing cooperative learning as the main pedagogical approach for at least one school year. 2.513 secondary education students (1.308 males, 1.205 females), 12–17 years old (*M* = 13.85, *SD* = 1.29), enrolled in 17 different schools belonging to the National Network of Schools on Cooperative Learning in Spain agreed to participate. They all had experienced this pedagogical approach a minimum of one school year. Participants were asked to complete the cooperative learning questionnaire, the strategies to control the study questionnaire and the global academic self-efficacy questionnaire. Participants were grouped based on their perceptions on cooperative learning and self-regulated learning in their classes. A combination of hierarchical and *κ*-means cluster analyses was used. Results revealed a four-cluster solution: cluster one included students with low levels of cooperative learning, self-regulated learning and academic self-efficacy, cluster two included students with high levels of cooperative learning, self-regulated learning and academic self-efficacy, cluster three included students with high levels of cooperative learning, low levels of self-regulated learning and intermediate-low levels of academic self-efficacy, and, finally, cluster four included students with high levels of self-regulated learning, low levels of cooperative learning, and intermediate-high levels of academic self-efficacy. Self-regulated learning was found more influential than cooperative learning on students’ academic self-efficacy. In cooperative learning contexts students interact through different types of regulations: self, co, and shared. Educators should be aware of these interactions, symmetrical or asymmetrical, because they determine the quality and quantity of the students’ participation and achievements, and they are key elements to prevent school failure.

## Introduction

Research has showed that individuals are able to monitor, control and regulate their behaviors in learning contexts, but all depends on the resources and the pedagogical approach used by the educators (Agina et al., 2011). Students’ active role in their own learning process begins very early, and continues along their lifetime (Zimmerman, 1989). Several elements have been identified as fundamental in this growth: cognition, metacognition, motivation, behavior and context (Pintrich, 2000; Dembo et al., 2006). Among them, context is considered a key factor to promote or mislead self-regulated learning (Agina et al., 2011). This concept refers to a “proactive process that students use to acquire academic skills, such as setting goals, selecting and developing strategies, and self-monitoring one’s effectiveness” (Zimmerman, 2008, p. 166).

Individuals are usually focused on regulating their own knowledge and behavior, with no intentions of influencing other students. Therefore, it is considered intra-personal (Grau and Whitebread, 2012). However, students are constantly challenged to work in pairs to learn. In this case, individuals must move into the inter-personal concept of regulation, *co-regulation*, which “means regulation directed toward a specific member of a group in a collective activity” (Hayes et al., 2015, p. 3). Students are forced to work with a class-mate (on a one-to-one basis) and interact with him/her to solve a learning task. Finally, students are also faced with cooperative and/or collaborative learning contexts where they have to relate with several other students to learn. *Shared-regulation* is referred as “processes by which multiple others regulate their collective activity” (Hadwin and Oshige, 2011, p. 254). In this context, group members “collectively set goals, track their progress, use strategies, and consider their effectiveness in the service of a shared outcome” (Hayes et al., 2015, p. 3). There is general consensus of the efficacy of self-regulated learning on academic success (Pintrich, 2000; Winne, 2005; Zimmerman, 2008). The question that this research brings is: how do cooperative learning contexts affect students’ self-regulated learning?

Cooperative learning has been associated to the development of cognitive, metacognitive and motivational skills in students, which can promote self-regulated learning (Efklides, 2008; Järvelä et al., 2008; Arjanggi and Setiowati, 2014). Among its basic elements positive interdependence can be considered very significant (the other ones are: promotive interaction, individual accountability, group processing, and interpersonal skills). It refers to the idea that students must help each other learn, because one’s success is dependent on others’ success (Johnson and Johnson, 2014). This is the basis of cooperative learning, but what types of relations are established between group members in this context? Does individuals’ personality influence them? Chamorro-Premuzic et al. (2007) believe that students’ personality traits have an effect on their learning in cooperative contexts. Individuals’ personality has been organized around five basic dimensions (Goldberg, 1990): extraversion (i.e., sociable, active), neuroticism (i.e., anxious, pessimistic), openness to experience (i.e., imaginative, curious), agreeableness (i.e., empathic, compassionate), and conscientiousness (i.e., organized, hard-working). The dimension extraversion/introversion has been associated to a preference for cooperative learning (Ramsay et al., 2000), but all of them can play a role in this type of contexts.

Within cooperative learning groups, the regulation processes can shift from one person providing all the information and adopting a leading instructional role, to a more co-regulatory and balanced situation where different group members provide information and instruction (Salonen et al., 2005). Moreover, within a group, there are individuals who adopt active roles (more participative), while others adopt passive roles (less participative). Unfortunately, many times, the most active ones are not always the better qualified, but they can become the most influential (Menges and Svinicki, 1991). Therefore, at times, cooperative learning contexts can negatively affect individuals’ self-regulated learning. Learn to learn is important, but also learn to cooperate. For researchers and scholars, cooperative learning is considered a pedagogical approach capable of successfully promote academic achievement (Johnson and Johnson, 2014; Slavin, 2014). The question that this study brings is: do all students in a cooperative learning context improve their self-regulated learning skills? And how both influence academic self-efficacy?

Self-regulated learning has long been associated to self-efficacy (Schunk, 1990; Zimmerman, 1990), since the first one depends on personal perceptions of efficacy, among other things. Self-efficacy has been defined as the belief in one’s ability to conduct the actions needed to achieve one’s goals (Bandura, 1997). Learners high on self-regulation, both high and low-achieving, tend to exhibit a high sense of efficacy in their own capabilities (Duckworth et al., 2009). In this same trend, one of the three motivational components with the highest influence on academic achievement is considered to be self-efficacy. It refers to the beliefs about one’s capacity to perform a class task. Its influence on students’ motivation is so important that it is considered the most powerful predictor of academic performance, effort and persistence (Pintrich and Schunk, 2002). Therefore, schools should try to improve both, self-regulation and self-efficacy, to prevent school failure, because every student needs to feel the support to develop the belief that he/she can improve his/her knowledge and skills and learn.

Self-efficacy, among other elements, can help at risk students overcome their at-risk conditions and have a positive impact in their academics (Cooper, 2015). School failure or individual’s progress in school have been related to different factors such as child characteristics, family background and contextual factors (i.e., school, teachers...) (Blair and Raver, 2015). Historically, they have been associated to general intelligence (Duckworth and Carlson, 2013). Not until recently, researchers have turned their eyes to a dimension of temperament linked to success in school: self-regulation (Blair and Raver, 2015). Learning to organize information and to engage in goal-directed tasks, to focus and maintain attention, to reflect on information and experience, to regulate emotions and to engage in positive social interactions have been shown instrumental to prevent school failure (Blair and Razza, 2007; McEwen and Gianaros, 2011; Blair and Raver, 2015).

As mentioned above, cooperative learning contexts demand students to self, co, and share-regulate their learning, and not all students know how to do it. Moreover, Leinonen et al. (2003) identified four types of knowledge construction based on students’ interactions: (i) active co-construction: these individuals frequently bring information to the group, actively collaborating with others; (ii) non-active co-construction: these individuals less frequently bring information to the group, but they access other’s information; (iii) comment receiver: these individuals receive information from others, providing feedback; and (iv) isolate receiver: these individuals receive little information from others with no reciprocal interaction. Therefore, students in cooperative learning groups play different roles to regulate their and others’ knowledge. Moreover, in these groups, individuals’ self-efficacy can significantly impact their feelings of collective efficacy (Fernandez-Ballesteros et al., 2002), influencing the group’s functioning and achievements. The question that this research brings is: how do cooperative learning, self-regulation and academic self-efficacy relate to each other? And how they connect to have an effect on students’ at risk of academic failure?

Previous research works have studied the interactions between self-regulation and self-efficacy (Schunk, 1990; Zimmerman, 1990), between self-regulation and cooperative learning (Arjanggi and Setiowati, 2014), and between self-efficacy and collaborative learning (Wang and Lin, 2007). However, no previous works have assessed the three elements at the same time.

**FIGURE 1:**
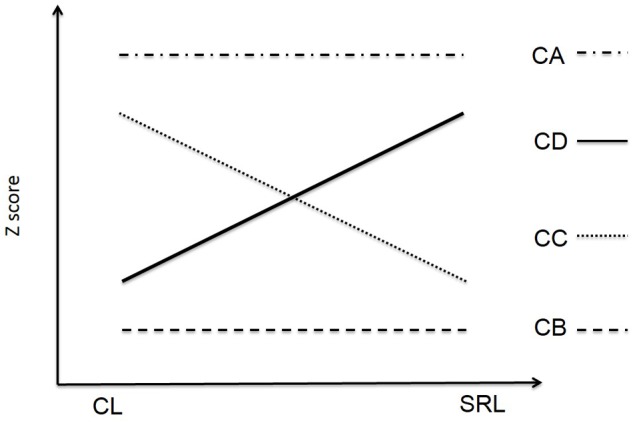
**Students’ groups in the initial hypothesis**.

**FIGURE 2:**
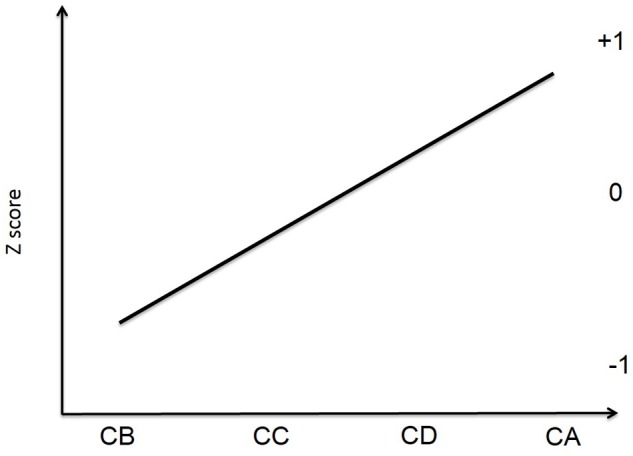
**Hypothesized relationship between academic self-efficacy and students’ groups**.

Based on the aforementioned, the main goal of the present study was to assess the interactions between self-regulated learning, cooperative learning and academic self-efficacy in secondary education students experiencing cooperative learning as the main pedagogical approach for at least one school year. The initial hypothesis was that some students will perceive high levels of self-regulated learning and cooperative learning (**Figure 1**, CA), other students will perceive low levels of both variables (**Figure 1**, CB), a third group will show high levels of cooperative learning and low levels of self-regulation (**Figure 1**, CC), and a fourth group will show high levels of self-regulated learning and low levels of cooperative learning (**Figure 1**, CD). A second hypothesis was that these groups will show different levels of academic self-efficacy: the higher the students’ self-regulation, the higher their self-efficacy; this means that group B will show the lowest scores on both variables, followed by groups C, D, and A (**Figure 2**).

## Materials and Methods

### Participants

2.513 secondary education students (1.308 males, 1.205 females), 12–17 years old (*M* = 13.85, *SD* = 1.29), enrolled in 17 different schools belonging to the National Network of Schools on Cooperative Learning in Spain agreed to participate. The main goal of this network is to use this methodology on a daily basis as one of its pillars. 411 students were considered at risk of academic failure (they had low grades in at least three school subjects), and 71 were immigrants. All participants had experienced cooperative learning a minimum of one school year. Based on the accessibility of their teachers, schools selected different subjects to implement this pedagogical approach (i.e., Maths, History, Science, Literature, Arts, Music, and Physical Education). They had to use, at least, one cooperative learning technique a week in their classes; for example: Think-Pare-Share (Kagan, 1992), Collective Score (Orlick, 1978), Student-Teams-Achievement-Division (Slavin, 1990), Learning Together (Johnson and Johnson, 1987), Co-op Co-op (Kagan, 1992), or Jigsaw (Aronson, 2010).

### Instruments

#### Cooperative Learning

The Cooperative Learning Questionnaire (Fernandez-Río et al., 2017), validated for secondary education and baccalaureate students, was used. It includes five subscales (four items each): interpersonal and small group skills (i.e., “We listen to groupmates’ ideas and perspectives”), Group processing (i.e., “Ideas are discussed among group members”), Positive interdependence (i.e., “My groupmates’ help is important to finish the tasks), Promotive interaction (i.e., “Group members interact during tasks”), and Individual accountability (i.e., “Each group member must participate in the tasks”). The following stem was added: “In class...” Each item was rated in a five-point likert scale from 1 “*corresponds not at all”* to 5 “*corresponds exactly.”* Cronbach’s alphas were adequate (original scores are presented between quotation marks): interpersonal and small group skills = 0.77 (0.74), Group processing = 0.79 (0.75), Positive interdependence = 0.74 (0.72), Promotive interaction 0.81 (0.76), and Individual accountability 0.79 (0.79). A global cooperative learning factor can also be obtained from the questionnaire.

#### Self-Regulated Learning

The Strategies to Control the Study Questionnaire (Hernández and García, 1995) was used to assess participants’ self-regulated learning. It includes three subscales: prior to the study period or the learning task (seven items: i.e., “I divide the task in parts to make it easier”), during the study period or learning task (six items: i.e., “If there is something I don’t understand, I do not continue until I understand it”), and after the study period or learning task (four items: i.e., “I review the whole task to see if I have any mistakes”). Cronbach’s alphas can be considered adequate (original scores are presented in parenthesis): prior to the study period or the learning task = 0.85 (0.82), during the study period or learning task = 0.72 (0.73), and after the study period or learning task = 0.78 (0.79).

#### Academic Self-Efficacy

The Global Academic Self-Efficacy Questionnaire (Torre, 2006) was used. It is a nine-item, one factor instrument (i.e., I feel that I have the capacity to pass all the subjects this year”). It has been validated for university students (Cronbach’s α = 0.90). In our study, it was validated for secondary education students, and it obtained an adequate Cronbach’s α = 0.90.

### Procedure

Project implementation involved several steps. Prior to data collection an informed written consent, approved by the researchers’ university Ethical Committee, was signed by all participants’ parents. Schools’ administrators were contacted to fully explain the research project. Near the end of the school year, each school was informed of all the necessary procedures to guarantee adequate data collection. In one session (45 min), all participants were granted access to the questionnaire through an online link provided by the research team (it was “open” only during 1 week for all the schools to obtain data at the same time of the year). Participating students were informed that their participation was voluntary, data obtained would be kept confidential, and it will not affect their grades. To minimize the tendency of participants to provide socially desirable answers, they were asked to be totally honest, guarantying complete anonymity and confidentiality. School administrators and not teachers were in charge of data collection to avoid any influence on the students’ responses. Questionnaire completion lasted an average of 20–25 min.

### Statistical Analyses

First, two confirmatory factor analyses were conducted using the program EQS 6.2. (Bentler, 2006). The first one produced a self-regulated learning index from the self-regulated learning questionnaire. The second one validated the academic self-efficacy questionnaire in secondary education students. Since preliminary data showed a substantial multivariate kurtosis, analyses were based on the Satorra-Bentler scaled chi-square statistic (S-Bχ^2^; Satorra and Bentler, 1988). The sample’s goodness-of-fit was performed using multiple criteria (Byrne, 2008): the Comparative Fit Index (^∗^CFI; Bentler, 1990), the Root Mean-Square Error of Approximation (^∗^RMSEA; Browne and Cudeck, 1993), and the Standardized Root Mean Square Residual (SRMR). The ^∗^CFI represents the CFI robust version calculated on the S-Bχ^2^ statistical basis. It is ranged from 0 to 1.00. Hu and Bentler (1999) suggested a value of 0.95 as indicative of good model fit. The ^∗^RMSEA is considered the robust version of the RMSEA and it considers the population’s approximation error (Browne and Cudeck, 1993). ^∗^RMSEA’s discrepancy is expressed in degrees of freedom, and it is sensitive to the model’s complexity. Values lower than 0.05 indicate a good fit, and values as high as 0.08 represent reasonable errors of approximation. To complete the analysis, the 90% confidence interval provided by the ^∗^RMSEA was also included (Steiger, 1990). Finally, the SRMR is the average standardized residual value. It is derived from fitting the hypothesized variance covariance matrix to the sample data. Its values range from 0 to 1.00. Values lower than 0.08 indicate a proper fit to the model (Hu and Bentler, 1999).

Second, descriptive and correlational analyses were conducted. To assess the initial hypotheses, participants were grouped based on their perceptions on cooperative learning and self-regulated learning in their classes. A combination of hierarchical and *κ*-means cluster analyses was used in different steps: (1) to identify the number of clusters and provide the necessary information for the next analysis (*k-*means), a hierarchical cluster analysis using Ward’s method and the squared Euclidian distance was conducted (variables’ scores were standardized using z-transformation; Huberty et al., 2005); (2) a *κ*-means cluster analysis was conducted in the groups obtained in the previous step to find the final cluster solution; and (3) this solution’s stability was tested and re-examined on a random sample (50%) of the total number of participants. In addition, Cohen’s *κ* was used to measure the degree of agreement (stability) of the subjects’ classification using data from the entire sample and the subsamples. Differences among clusters in all behavioral variables were estimated using analysis of variance *post hoc* Tukey’s HSD test.

## Results

Confirmatory factor analyses showed a good fit to the model (**Table 1**). **Table 2** shows means and standard deviations of all variables. Cooperative learning and self-regulated learning’s levels were similar. Correlations between these variables and academic self-efficacy were positive and significant (*p* < 0.001), and the highest one was found between self-regulated learning and academic self-efficacy. Cronbach’s alphas were also very high in all variables (≥0.90).

**Table 1:** Confirmatory factor analyses.

	S-Bx2	*df*	^∗^CFI	SRMR	^∗^RMSEA (90% CI)	
**Self-regulated learning**						
M_1_	434.75	116	0.971	0.035	0.033	(0.030–0.036)
**Academic self-efficacy**						
M_2_	170.35	27	0.978	0.024	0.046	(0.039–0.053)

**Table 2:** Mean, standard deviation, and correlations among variables.

	α	*M*	*SD*	1	2	3
(1) Cooperative learning	0.92	3.72	0.65	1		
(2) Self-regulated learning	0.90	3.70	0.71	0.38^∗∗∗^	1	
(3) Academic self-efficacy	0.90	3.89	0.75	0.29^∗∗∗^	0.60^∗∗∗^	1

*^∗∗∗^*p* < 0.001.*

A second correlation analysis was conducted among all the cooperative learning subscales (interpersonal and small group skills, group processing, positive interdependence, promotive interaction, and individual accountability), all the self-regulated learning subscales (pre, in, and post) and academic self-efficacy. They all were positive and significant (*p* < 0.01). The highest ones were found between the different subscales of each scale, and also between academic self-efficacy and the different subscales of self-regulated learning (**Table 3**).

**Table 3:** Correlations among all subscales.

	1	2	3	4	5	6	7	8	9
(1) Interpersonal and small group skills	1								
(2) Group processing	0.77^∗∗^	1							
(3) Positive interdependence	0.55^∗∗^	0.58^∗∗^	1						
(4) Promotive interaction	0.60^∗∗^	0.63^∗∗^	0.65^∗∗^	1					
(5) Individual accountability	0.54^∗∗^	0.55^∗∗^	0.59^∗∗^	0.56^∗∗^	1				
(6) Pre-self-regulated learning	0.32^∗∗^	0.33^∗∗^	0.28^∗∗^	0.26^∗∗^	0.24^∗∗^	1			
(7) In-self-regulated learning	0.35^∗∗^	0.33^∗∗^	0.29^∗∗^	0.31^∗∗^	0.26^∗∗^	0.67^∗∗^	1		
(8) Post-self-regulated learning	0.28^∗∗^	0.28^∗∗^	0.23^∗∗^	0.21^∗∗^	0.22^∗∗^	0.64^∗∗^	0.66^∗∗^	1	
(9) Academic self-efficacy	0.29^∗∗^	0.28^∗∗^	0.18^∗∗^	0.25^∗∗^	0.21^∗∗^	0.53^∗∗^	0.56^∗∗^	0.49^∗∗^	1

*^∗∗^*p* < 0.01 (bilateral).*

Based on the cooperative learning and self-regulated learning factors, participants were grouped in clusters. An exploratory multivariate data reduction technique was used to place students into relatively homogenous groups, maximizing similarities within students belonging to a particular cluster and dissimilarities between students belonging to different clusters. Significant changes were observed from the two-cluster to the three-cluster solution, and from this one to the four-cluster solution. Therefore, three solutions with two, three and four clusters were considered. The two-cluster solution produced two groups: (a) high self-regulated learning and cooperative learning, and (b) low self-regulated learning and cooperative learning. The three-cluster solution produced three groups: the previous two, and (c) low self-regulated learning and high cooperative learning. Finally, the four-cluster solution produced four groups: (a) high self-regulated learning and cooperative learning; (b) low self-regulated learning and cooperative learning; (c) low self-regulated learning and high cooperative learning; and (d) high self-regulated learning and low cooperative learning. Significant differences were obtained among the four clusters in both grouping variables (*p* < 0.001). Balancing parsimony and explanatory power, the four-cluster solution was selected based on the following criteria: (i) the agglomeration coefficients yielded a relatively large change, (ii) statistically significant differences were identified between clusters; and (iii) differences among groups were more consistent from a theoretical and an empirical point of view. This solution’s stability was tested through a *k*-means cluster analysis in 50% of the original sample, randomly selected, and similar values were obtained (Kappa Cohen = 0.81; Landis and Koch, 1977).

**FIGURE 3:**
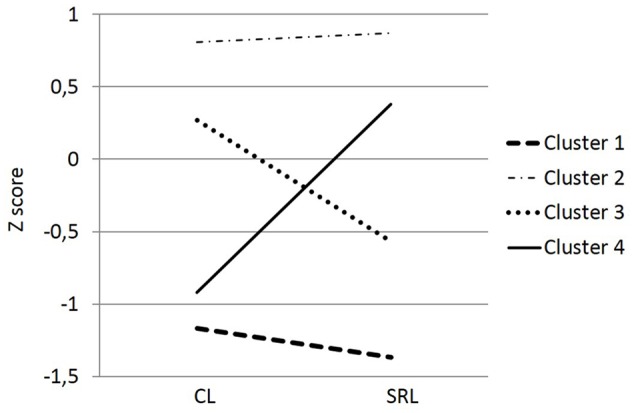
**The four clusters identified**.

**Figure 3** shows the four different groups identified, and **Table 4** presents their characteristics. Cluster one included 395 students with a low profile on both clustering variables: cooperative learning and self-regulation. The majority were males (64.1%), 25.36% were students “at risk of academic failure” (the highest), and 3.5% were immigrants. Cluster two included 888 students with a high profile on both variables. The majority were females (53.6%), 11.1% were students “at risk of academic failure” (the lowest percentage), and 2.5% were immigrants. Cluster three included 735 students with a profile high on cooperative learning and low on self-regulated learning. The highest percentage were males (53.6%), 20.7% were students “at risk of academic failure” (20.7%), and 2.9% were immigrants. Finally, cluster four included 495 students with a profile high on self-regulated learning and low on cooperative learning. It contained similar number of males (50.9%) and females, a low percentage of students “at risk of failure” (11.9%), and immigrants (2.8%).

**Table 4:** Clusters’ characteristics.

Clustering variable	Cluster 1 (*n* = 395)		Cluster 2 (*n* = 888)		Cluster 3 (*n* = 735)		Cluster 4 (*n* = 495)	
	Mean (z)	*SD*	Mean (z)	*SD*	Mean (z)	*SD*	Mean (z)	*SD*
(1) Cooperative learning	2.95ˆd (-1.17)	0.53	4.25ˆa (0.81)	0.35	3.90ˆb (0.27)	0.47	3.12ˆc (-0.92)	0.49
(2) Self-regulated learning	2.73ˆd (-1.37)	0.52	4.32ˆa (0.87)	0.33	3.30ˆc (-0.57)	0.51	3.97ˆb (0.38)	0.71
(3) Academic self-efficacy	3.24ˆd (-0.81)	0.83	4.30ˆa (0.55)	0.50	3.64ˆc (-0.33)	0.55	4.04ˆb (0.19)	0.75
**Characteristics**								
Males *n* (%)	253 (64.1%)		409 (46.1%)		394 (53.6%)		252 (50.9%)	
Females *n* (%)	142 (35.9%)		479 (53.9%)		341 (46.4%)		243 (49.1%)	
Risk of failure *n* (%)	101 (25.6%)		99 (11.1%)		152 (20.7%)		59 (11.9%)	
Immigrants *n* (%)	14 (3.5%)		22 (2.5%)		21 (2.9%)		14 (2.8%)	

*Means in the same row which do not share superscripts differ at *p* < 0.01.*

**FIGURE 4:**
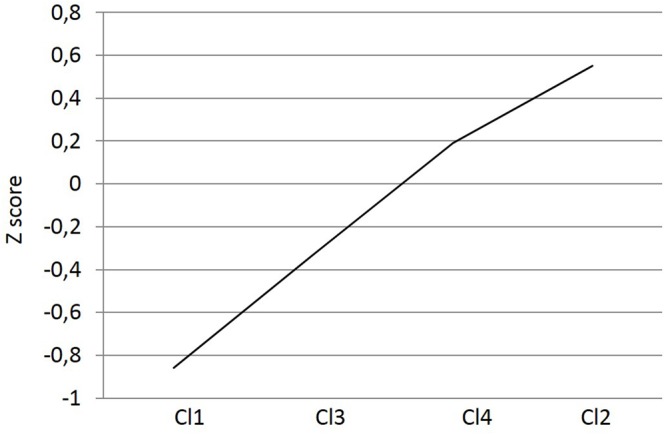
**Academic self-efficacy on each cluster**.

**FIGURE 5:**
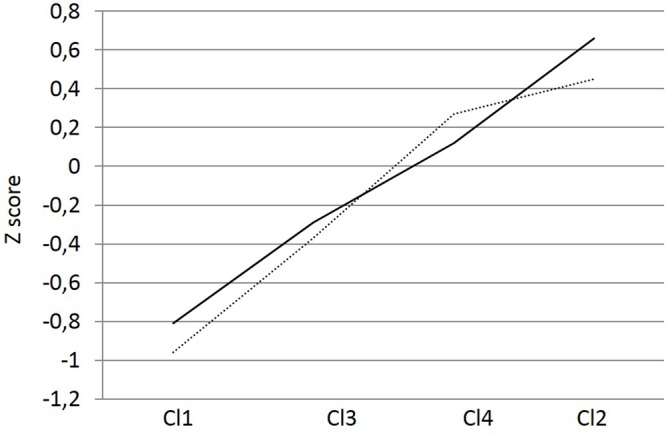
**Interaction cluster × gender**.

An univariate analysis of variance was conducted using academic self-efficacy as the dependent variable and cluster and gender as independent variables. A significant main effect emerged for cluster: *F*(3,2505) = 299.14, *p* < 0.001, η^2^ = 0.26, and its interaction with gender: *F*(3,2505) = 5.32, *p* < 0.01, η^2^ = 0.01. Tukey’s HSD *post hoc* tests were conducted to compare groups (**Figure 4**). Statistically significant differences (*p* < 0.001) were found among all groups (clusters) in academic self-efficacy. Finally, **Figure 5** shows the interaction cluster^∗^gender. In all clusters, males’ scores were higher than females, except in cluster four, where females scored higher.

## Discussion

The goal of the present study was to assess the interactions between self-regulated learning, cooperative learning and academic self-efficacy in secondary education students experiencing cooperative learning as the main pedagogical approach for at least one school year. The initial hypothesis was that one group of students will perceive high levels of self-regulated learning and cooperative learning, other group will perceive low levels of both variables, a third group will show high levels of cooperative learning and low levels of self-regulation, and a fourth group will show high levels of self-regulated learning and low levels of cooperative learning. A second hypothesis was that these groups will show different levels of academic self-efficacy: the higher the students’ self-regulation, the higher their self-efficacy; this means that group B will show the lowest, followed by groups C, D, and A. Results obtained support both hypothesis, and they revealed four clusters: cluster 1: low levels of cooperative learning, self-regulated learning and academic self-efficacy; cluster 2: high levels of cooperative learning, self-regulated learning and academic self-efficacy; cluster 3: high levels of cooperative learning, low levels of self-regulated learning and intermediate-low levels of academic self-efficacy, and cluster 4: high levels of self-regulated learning, low levels of cooperative learning, and intermediate-high levels of academic self-efficacy.

Regarding the first hypothesis, four groups of students were obtained, supporting it. Cluster 2 was the largest one (888 students) and it included students who perceived high levels of self-regulated, cooperative learning and academic self-efficacy. This is consistent with previous studies that showed that cooperative learning can foster cognitive, metacognitive, and motivational skills (Pintrich, 2000; Dembo et al., 2006), with those which observed that cooperative learning can promote self-regulated learning (Järvelä et al., 2008; Arjanggi and Setiowati, 2014) and with the ones which have linked self-regulation and self-efficacy (Pintrich and Schunk, 2002; Duckworth et al., 2009). Cluster 2 could be considered the most adaptive group, since students high on self-regulated learning have been found to be more proactive, and they tend to show initiative, persistence and adaptive skills, originated from positive metacognitive and motivational skills (Zimmerman, 2008). Plan, guide and monitor one’s personal conduct seem to allow individuals to self-regulate their participation in the cooperative work, increasing group processing, which can lead to improved results (Jermann and Dillenbourg, 2008; Mauri et al., 2009). Cooperative learning has been found effective when different perspectives were confronted. This can help activate different interactive processes: attention, metacognition, motivation, emotion, action, and volitional control (Boekaerts and Niemivirta, 2000; Boekaerts and Corno, 2005). When this happens, the context allows regulation among group members, which tends to favor self-regulated learning in all of them (Monereo, 2007). In the present study, data obtained in cluster 2 could be considered very positive: individuals in this group showed the highest levels of academic self-efficacy, cooperative learning and self-regulation and the lowest percentage of students at risk of academic failure (11.1%). Previous studies have showed that individuals’ self-efficacy can significantly impact a groups’ feelings of collective efficacy (Fernandez-Ballesteros et al., 2002), influencing its functioning and its achievements. Fortunately, it was the biggest group (888 students), which is consistent with the background of the targeted sample: schools belonging to the National Network of Schools on Cooperative Learning, who had been integrating this pedagogical model on a regular basis during the whole school year.

Opposite to the previous group, cluster 1 could be considered the least adaptive one. It included students who showed low levels of cooperative learning, self-regulated learning and academic self-efficacy. This is consistent with the findings of the previous group. Students who feel that the different features linked to self-regulation described in the previous paragraph do not refer to them are expected to produce opposite results. If these individuals feel that the educational context does not allow them to confront ideas or perspectives, it will not force these students to adapt and reach agreements, helping them regulate theirs and others’ behaviors. These students probably felt that the learning contexts did not allow for co and shared regulation, creating unbalanced situations where, maybe, only a few group members provided information and instruction, taking a dominant role in the group (Salonen et al., 2005). Students in this cluster probably felt that the relationships created in their groups were asymmetrical, affecting their self-regulation processes. These students’ probably behaved as isolated receivers of knowledge (received little information from others with no reciprocal interaction) (Leinonen et al., 2003). The end result could be considered very negative: the lowest levels of self-regulated learning, cooperative learning and academic self-efficacy. Fortunately, it was the smallest group (395 participants), which is also consistent with the background of the targeted sample: schools belonging to the National Network of Schools on Cooperative Learning. It was also the cluster with the highest percentage of students at risk of academic failure (25.6%). This is consistent with the scores obtained by these students in the other variables: low self-regulated learning and academic self-efficacy. These results are supported by previous studies which showed that group members’ personal self-efficacy beliefs can significantly impact the group’s feelings of collective efficacy (Fernandez-Ballesteros et al., 2002), influencing its functioning and its achievements. Educators should be aware that learning contexts that promote self-regulation imply choice and consistency (Sheldon and Elliot, 1998), “clarity and pace of instruction, student autonomy, teacher enthusiasm, humor, fairness, and teacher expectations about students’ capacity” (Boekaerts and Cascallar, 2006, p. 204). If teachers want to promote self-regulation in their students, they must create specific class structures to incorporate these ideas.

Cluster 3 included students who scored high on cooperative learning, but low on self-regulated learning, showing a negative correlation between both variables. The first impression is that cooperative learning seemed to distort student’s self-regulated learning. Previous studies have showed that cooperative learning can promote or hinder self-regulated learning, depending on the relations created among group members (Agina et al., 2011). Asymmetrical relations in working groups can lead to unbalanced instruction, failure in co-regulation, and negative feelings and behaviors among group members (Salonen et al., 2005). In cooperative learning contexts, some individuals adopt active roles (more participative), forcing other group members to adopt passive roles (less participative). These individuals tend to non-actively co-construct their knowledge, probably behaving as comment receivers (received information from others and providing feedback) or even isolated receivers (received little information from others with no reciprocal interaction) (Leinonen et al., 2003). The first students usually dominate the group, adopting the role of instructors, and hindering their groupmates’ self-regulatory processes (Menges and Svinicki, 1991; Ramsay et al., 2000). Results from the present study indicated that in cluster 3, group members’ roles were not balanced. Educators should be aware of the relations that can emerge among group members in cooperative learning contexts, because some of them can negatively affect students’ self-regulated learning and knowledge construction. They should behave as activators of the teaching-learning process and prevent asymmetrical relations among groupmates (Fernández-Río, 2016). When these appear, cooperative learning does not positively correlate to self-regulated learning. Results seem to indicate that this was the case in cluster 3, and the end result was negative: intermediate-low levels of academic self-efficacy. This cluster included the second highest percentage of students at risk of school failure (20.7%), which is consistent with the scores obtained in the other variables. Previous studies have showed that low levels of self-regulated learning tend to produce low levels of academic self-efficacy, which can lead to academic failure (Pintrich and Schunk, 2002).

The final cluster, number 4, included students with high levels of self-regulation and low levels of cooperative learning, showing a negative correlation between both variables. However, unlike in cluster 3, it produced high levels of academic self-efficacy. This is noteworthy, because it shows that self-regulated learning was more influential than cooperative learning on students’ academic self-efficacy (results also showed the highest correlation between these two variables). This is consistent with previous research works which showed that high levels of self-regulation have been linked to high levels of academic self-efficacy efficacy (Duckworth et al., 2009). Students in this cluster probably behaved as active co-constructors of knowledge (providing large amounts of information to the groupmates), non active co-constructors of knowledge (providing some information to the groupmates) and/or comment receivers (receiving information from others and providing feedback) (Leinonen et al., 2003). Their scores indicated that they perceived low levels of cooperative learning in their groups, and consequently, asymmetrical relationships among group members. They probably thought that other group members did not cooperate, becoming active and dominant or passive or non-dominant members; in both cases taking advantage of the work of others. In any case, they showed high levels of self-regulated learning. The end result could be considered positive: intermediate-high levels of academic self-efficacy. This cluster included the second lowest percentage of students at risk of school failure (11.9%), which is consistent with the scores obtained in the other variables. Previous studies indicated that low achieving students can also show high levels of self-efficacy (Duckworth et al., 2009), they just need help form the teachers to avoid school failure and the different issues associated (Cooper, 2015).

Regarding the second hypothesis, results showed that the higher the students’ self-regulation, the higher their academic self-efficacy. Cluster 2 scored higher in both variables, followed by cluster 4, cluster 3, and cluster 1. The same trend was observed in the correlational analyses: the highest score was obtained between these two variables, both when they were assessed globally (**Table 2**), and when the three subscales of self-regulated learning (pre, in, and post) were used in the analysis (**Table 3**). As previously mentioned, preceding studies have showed that learners high on self-regulation, both high and low-achieving, tend to exhibit high feelings of effectiveness in their own capabilities (Duckworth et al., 2009). Teachers can help their students develop self-regulation skills and have a positive impact in their self-efficacy showing them that they must: orient themselves before starting a task, collect relevant resources, integrate different viewpoints, monitor for comprehension and assess one’s progress (Boekaerts and Cascallar, 2006). Teachers must also help all students learn to persist on the class’ tasks, to work to overcome the difficulties that they face daily, to invest enough effort to be successful, and to try increasingly demanding tasks. If teachers focus on these ideas their students will develop their self-efficacy and, consequently, it will have an impact in the students’ self-regulation or vice versa. It is an extremely important goal and schools should try to improve both skills in all their students to prevent school failure.

Finally, results also showed significant differences in the interaction between clusters and gender. Males scored higher in all cluster except cluster 4. To our knowledge, there are no published studies that have addressed this connection to compare our results.

## Conclusion

Students experiencing cooperative learning as the main pedagogical approach model for at least one school year were grouped in four clusters: (1): low levels of cooperative learning, self-regulated learning and academic self-efficacy; (2): high levels of cooperative learning, self-regulated learning, and academic self-efficacy; (3): high levels of cooperative learning, low levels of self-regulated learning, and intermediate-low levels of academic self-efficacy; and (4): high levels of self-regulated learning, low levels of cooperative learning, and intermediate-high levels of academic self-efficacy. Self-regulated learning was found more influential than cooperative learning on students’ academic self-efficacy. In cooperative learning contexts students interact through different types of regulations: self, co, and shared. Educators should be aware of these interactions, symmetrical or asymmetrical, because they determine the quality and quantity of their participation and their achievements, and they are key elements to prevent school failure.

The present study also holds some limitations. First, its cross-sectional design does not allow to establish any causal relationship between the variables assessed. Longitudinal studies should assess the impact of purposely designed interventions. Second, the participants’ cooperative learning exposure was not fully controlled. All students had a minimum of 1 year experience, but the numbers of hours per week or years that the students have been following this method were not considered. Future studies should assess the impact of different hours, academic subjects, and techniques.

## Ethics Statement

The study was carried in accordance with the recommendations of the University of Oviedo Ethics Committee with written informed consent from all participants. All subjects gave written informed consent in accordance with the Declaration of Helsinki. The protocol was approved by the University of Oviedo Ethics Committee.

## Author Contributions

JF-R: study design, manuscript preparation; JC: study design, statistical analysis; AM-G: study design; DM-A: study design, data collection; JP: study design, data collection.

## Conflict of Interest Statement

The authors declare that the research was conducted in the absence of any commercial or financial relationships that could be construed as a potential conflict of interest.
